# Hospitalization information and burden of pediatric inpatients in transport accidents

**DOI:** 10.1186/s12889-024-18891-2

**Published:** 2024-05-30

**Authors:** Jing Yu, Lin Mei, Yanni Wang, Guoshuang Feng, Yueping Zeng, Xin Xu, Xinyu Wang, Jing Liu

**Affiliations:** 1 Department of Burn and Plastic Surgery, Beijing Children’s Hospital, Capital Medical University, National Center for Children’s Health, 56 Nanlishi Rd, Xicheng, Beijing, 100045 China; 2 Department of Otolaryngology, Head and Surgery, Beijing Children’s Hospital, Capital Medical University, National Center for Children’s Health, Beijing, 100045 China; 3 Big Data Center, Beijing Children’s Hospital, Capital Medical University, National Center for Children’s Health, 56 Nanlishi Rd, Xicheng, Beijing, 100045 China; 4 Medical Record Management Office, Beijing Children’s Hospital, Capital Medical University, National Center for Children’s Health, Beijing, 100045 China; 5 Information Center, Beijing Children’s Hospital, Capital Medical University, National Center for Children’s Health, Beijing, 100045 China

**Keywords:** Hospitalization information, Hospitalization burden, Pediatric, Traffic accidents

## Abstract

**Background:**

Transport accidents are one of the leading causes of child morbidity and mortality worldwide and represent a significant public health burden. This study aimed to investigate the hospitalization information and burden of pediatric inpatients in transport accidents in China.

**Methods:**

In this study, we collected the cover page of the medical records of pediatric inpatients in transport accidents using the Futang Research Center of Pediatric Development (FRCPD) database from January 1, 2016 to December 31, 2021. Then, we extracted the epidemiological characteristics, including demographic characteristics, cases distribution, disease information, and hospitalization burden.

**Results:**

Among 36,455 included inpatients, males, aged 1–3 years, East China, July were dominant in different subgroups. In transport accidents, pedestrians were the most frequently type of injury (65.69%). Of all known lesions, craniocerebral/nerve injury was the more common results in pediatric inpatients in transport accidents (33.93%). In addition to pedal cyclists more susceptible to sport system injury, other types of injured person with transport accidents were mainly craniocerebral/nerve injury. In terms of the type of discharge, occupant of heavy transport vehicle or bus and people with craniocerebral/nerve injury had the highest mortality rate after hospitalization in all type and lesion of injured person groups, respectively. The largest hospitalization burden in the type of injured person was occupant of heavy transport vehicle or bus.

**Conclusions:**

This study revealed that epidemiological characteristics and the main factor influencing the hospitalization information and burden of children with traffic accidents in China.

**Supplementary Information:**

The online version contains supplementary material available at 10.1186/s12889-024-18891-2.

## Introduction

Transport accidents are one of the leading causes of child morbidity and mortality worldwide and represent a significant public health burden. According to the World Health Organization (WHO)’s 2018 Global Status Report on Road Safety, more than 10 million children are injured in transport accidents every year, and up to 186,300 children lose their lives [1]. In China, transport accidents are the second leading cause of injury and death among children. A previous report showed that in 2019, 19.619 children under the age of 15 were injured and 2,593 were killed in transport accidents [2]. Faced with severe transport accidents problems, children are the vulnerable groups that need special attention in road traffic safety. However, as a country with a huge number of children, China does not have adequate epidemiological information about the pediatric inpatients in transport accidents.

The Futang Research Center of Pediatric Development (FRCPD) is a large medical consortium that promotes the development of children’s medical research [3]. It covers 47 tertiary children’s hospitals, 28 of which have agreed and uploaded the annual summary reports of discharged patients since 2016 in China. Herein, we retrospectively analyzed the epidemiological characteristics of pediatric inpatients in transport accidents using the FRCPD database, aiming to investigate the current situation of transport accidents among children in China and carry out targeted intervention activities.

## Methodology

### Data source and categories

The FRCPD was established as a multi-tiered pediatric diagnosis and treatment network. The data uploading docking and format standards are referred to the requirements made by the Hospital Quality Monitoring System (HQMS) for the collection of first-page information of inpatient medical records in the performance appraisal and medical quality management of national tertiary public hospitals (2019). In this study, the data came from the cover page of the medical records of discharged children using FRCPD database from January 1, 2016 to December 31, 2021. Detailed information regarding the FRCPD is learned at http://www.futang.org/about/fu-tang-jie-shao.htm. The inclusion criteria & exclusion criteria were shown in Table 1.

**Table 1:** Rules for inclusion criteria and exclusion criteria

Inclusion criteria	V01-V09: Pedestrian injured in transport accident
	V10-V19: Pedal cyclist injured in transport accident
	V20-V29: Motorcycle rider injured in transport accident
	V30-V39: Occupant of three-wheeled motor vehicle injured in transport accident
	V40-V49: Car occupant injured in transport accident
	V50-V59: Occupant of pick-up truck or van injured in transport accident
	V60-V69: Occupant of heavy transport vehicle injured in transport accident
	V70-V79: Bus occupant injured in transport accident
	V80-V99: Others
**Exclusion criteria**	The length of Hospitalization < 1 day
	The hospitalization charge is less than 5 yuan
	Incomplete important information, such as age, gender, and primary diagnosis.

Next, we extracted the epidemiological characteristics of pediatric inpatients in transport accidents, including demographic characteristics (gender, age), cases distribution (region, month of hospitalization), disease information (type/lesion of injured person, type of discharge), hospitalization burden (length of hospital stay/LOS and hospitalization expenses). Then, we divided age of hospitalization into five categories: < 1 years old, 1–3 years old, 4–6 years old, 7–12 years old, and 13–18 years old. Then, the lesion of injured person was summarized into five groups including craniocerebral/nerve injury, sports system injury, visceral-related injury, skin injury, and others. Next, the type of discharge was divided into discharge with doctor’s advice, discharge without doctor’s advice, death, transfer with doctor’s advice, transfer to community health service agencies/township health center with doctor’s advice, others. Furthermore, this 28 provincial and municipal hospitals were respectively belong to Northeast, North, East, Northwest, Southwest, South, and Central China regions (Sup Table 1).

### Statistics

Categorical variables, including gender, age, region, month of hospitalization, type/lesion of injured person, type of discharge were expressed as numbers (N), and were compared among different groups using the Pearson chi-square tests. Continuous variables including LOS and hospitalization expenses, were both non-normally distributed based on the Shapiro-Wilk tests. Therefore, they were expressed as median and interquartile ranges, and compared using Kruskal-Wallis tests. Post-hoc tests were conducted using the Steel-Dwass method. *P* < 0.05 was assessed as statistically significant. Statistical analyses were conducted with the JMP Pro 15 software.

## Results

### Demographic characteristics and cases distribution of pediatric inpatients in transport accidents

Firstly, over 7 million cover pages of the medical records were screened from the FRCPD database, and 36,455 pediatric inpatients in transport accidents were included in this study. As shown in Table 2, the included males accounted for 62.78% (*n* = 22,886), and females 37.22% (*n* = 13,569), with a male-female ratio of 1.68:1. The results showed that 1–3 years old inpatients were dominant (*n* = 12,258, 33.63%), followed by 4–6 years old (*n* = 11,388, 31.24%), 7–12 years old (*n* = 9970, 27.35%), 13–18 years old (*n* = 1698, 4.66%), and < 1 years old (*n* = 1141, 3.13%) (Table 2).

**Table 2:** The baseline characteristics of pediatric inpatients in transport accidents

Categories	Pediatric inpatients
**SUM**	36,455
**Gender**	
Male	22,886(62.78%)
Female	13,569(37.22%)
**Age**	
< 1 years old	1141(3.13%)
1–3 years old	12,258(33.63%)
4–6 years old	11,388(31.24%)
7–12 years old	9970(27.35%)
13–18 years old	1698(4.66%)
**Region**	
Northeast China	1475(4.05%)
North China	10,258(28.14%)
East China	15,080(41.37%)
South China	1525(4.18%)
Central China	2920(8.01%)
Northwest China	3504(9.61%)
Southwest China	1693(4.64%)
**Type of injured person**	
Pedestrian	23,949(65.69%)
Pedal cyclist	3508(9.62%)
Motorcycle rider or occupant of three-wheeled motor vehicle	2782(7.63%)
Occupant of car, pick-up truck or van	1867(5.12%)
Occupant of heavy transport vehicle or bus	89(0.24%)
Others	4260(11.69%)
**Lesion of injured person**	
Craniocerebral/nerve	12,370(33.93%)
Sports system	9615(26.37%)
Visceral-related	3524(9.67%)
Skin	3230(8.86%)
Others	7716(21.17%)
**Type of discharge**	
Discharge with doctor’s advice	32,907(90.27%)
Discharge without doctor’s advice	2966(8.14%)
Death	171(0.47%)
Transfer with doctor’s advice	73(0.20%)
Transfer to community health service agencies/ township health center with doctor’s advice	29(0.08%)
Others	309(0.85%)

**Fig. 1:**
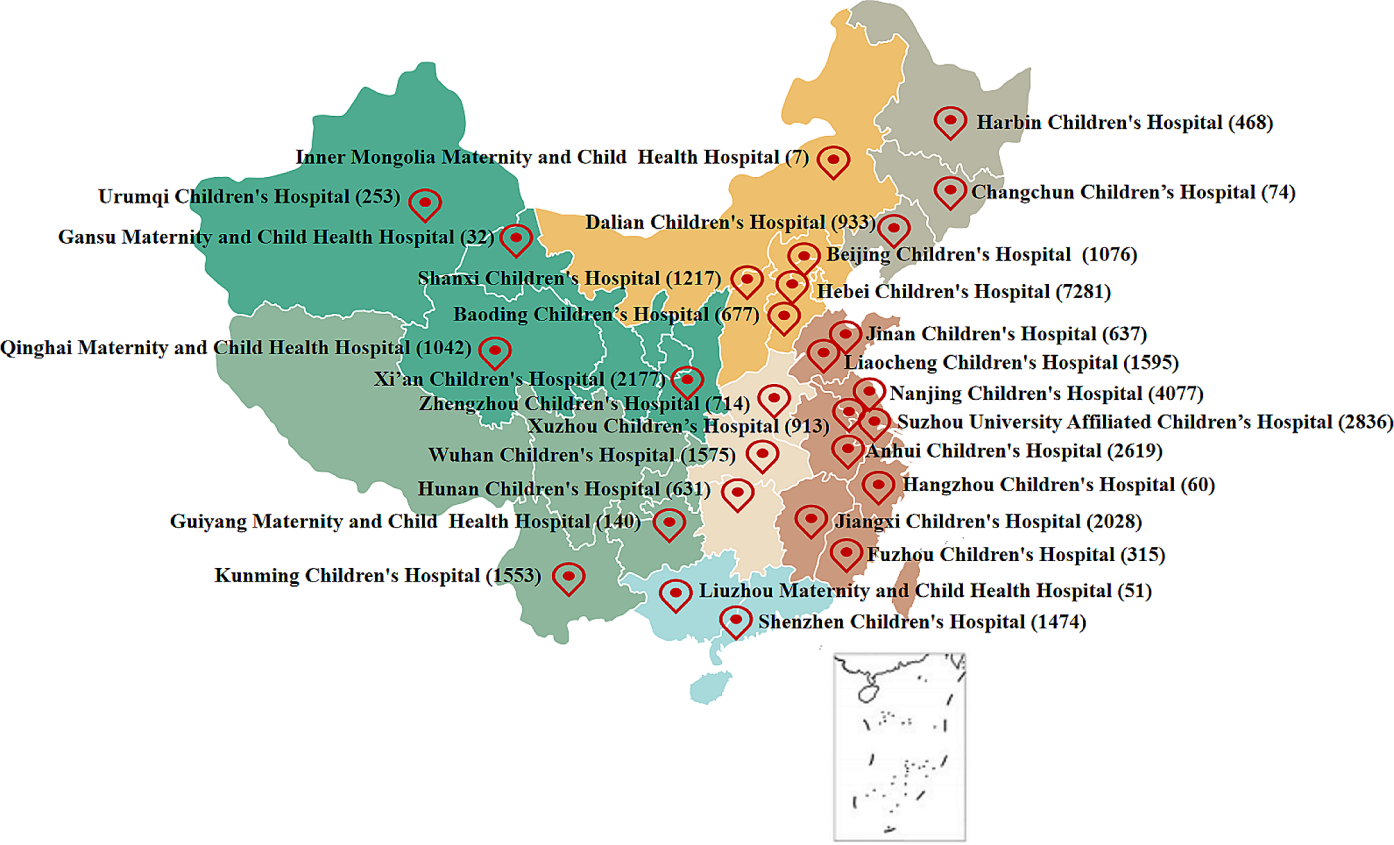
The region and hospital distribution of pediatric inpatients in traffic accidents

**Fig. 2:**
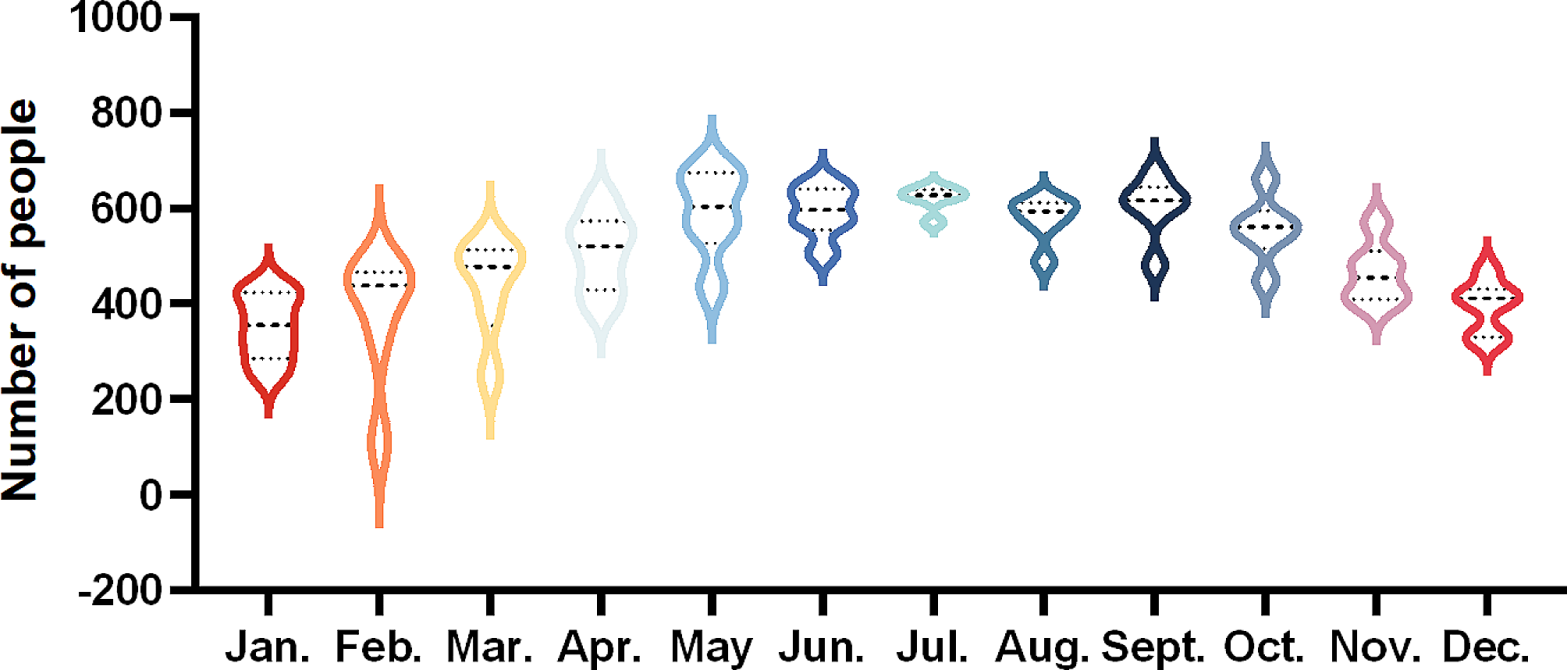
Total number of pediatric inpatients in traffic accidents per month, 2016 to 2021

Since this 28 hospitals of FRCPD database belonged to seven different regions in China, Fig. 1; Table 2 revealed that the top 3 regions with the highest numbers of patients with transport accidents were East China (*n* = 15,080, 41.37%), North China (*n* = 10,258, 28.14%), and Northwest China (*n* = 3504, 9.61%). Basing the month of hospitalization, the proportion of patients with transport accidents in July was largest (Fig. 2 and Sup Table 2).

### Disease information of pediatric inpatients in transport accidents

**Fig. 3:**
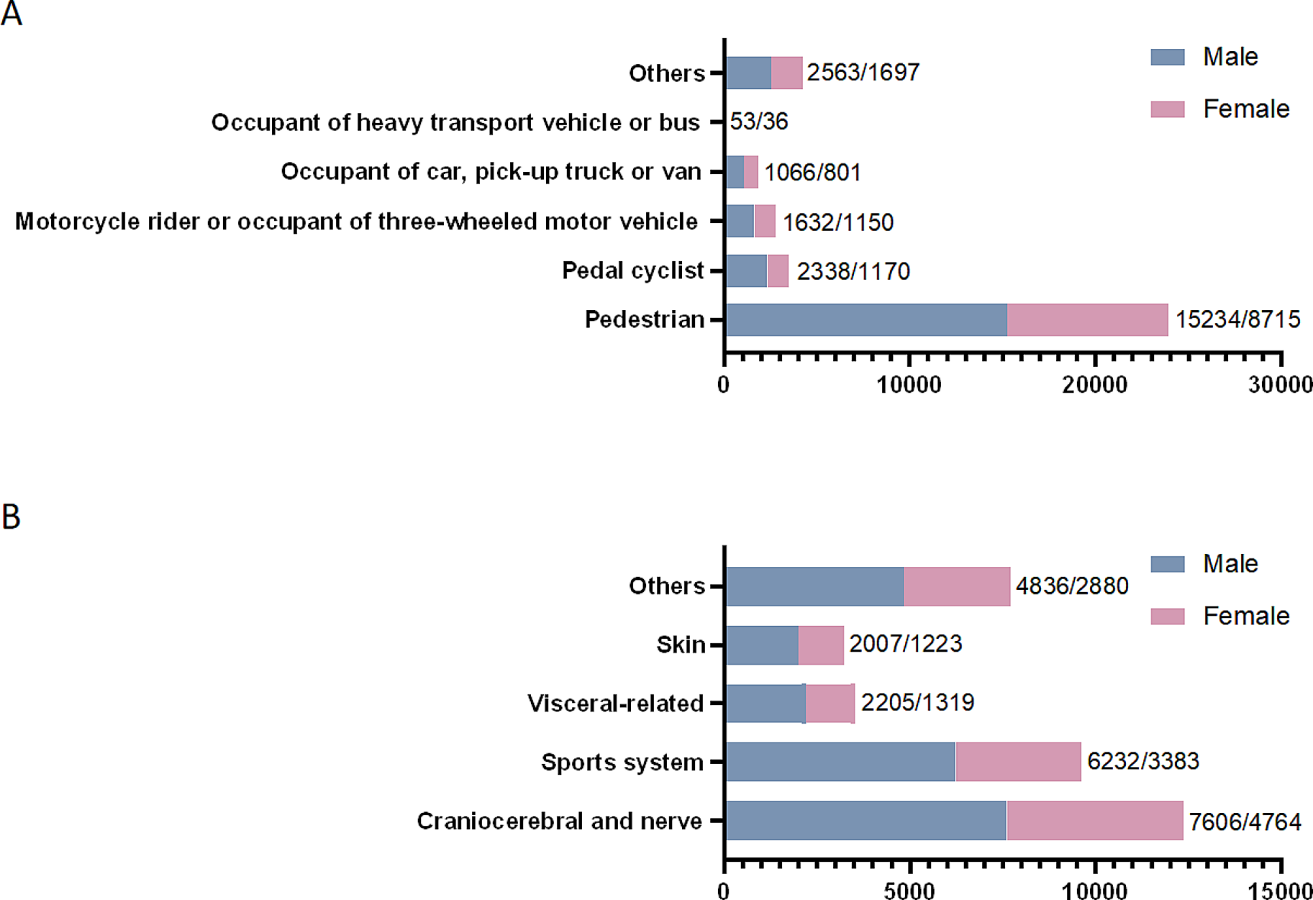
Gender distribution of pediatric inpatients in different type (A) and lesion (B) of injured person groups

**Fig. 4:**
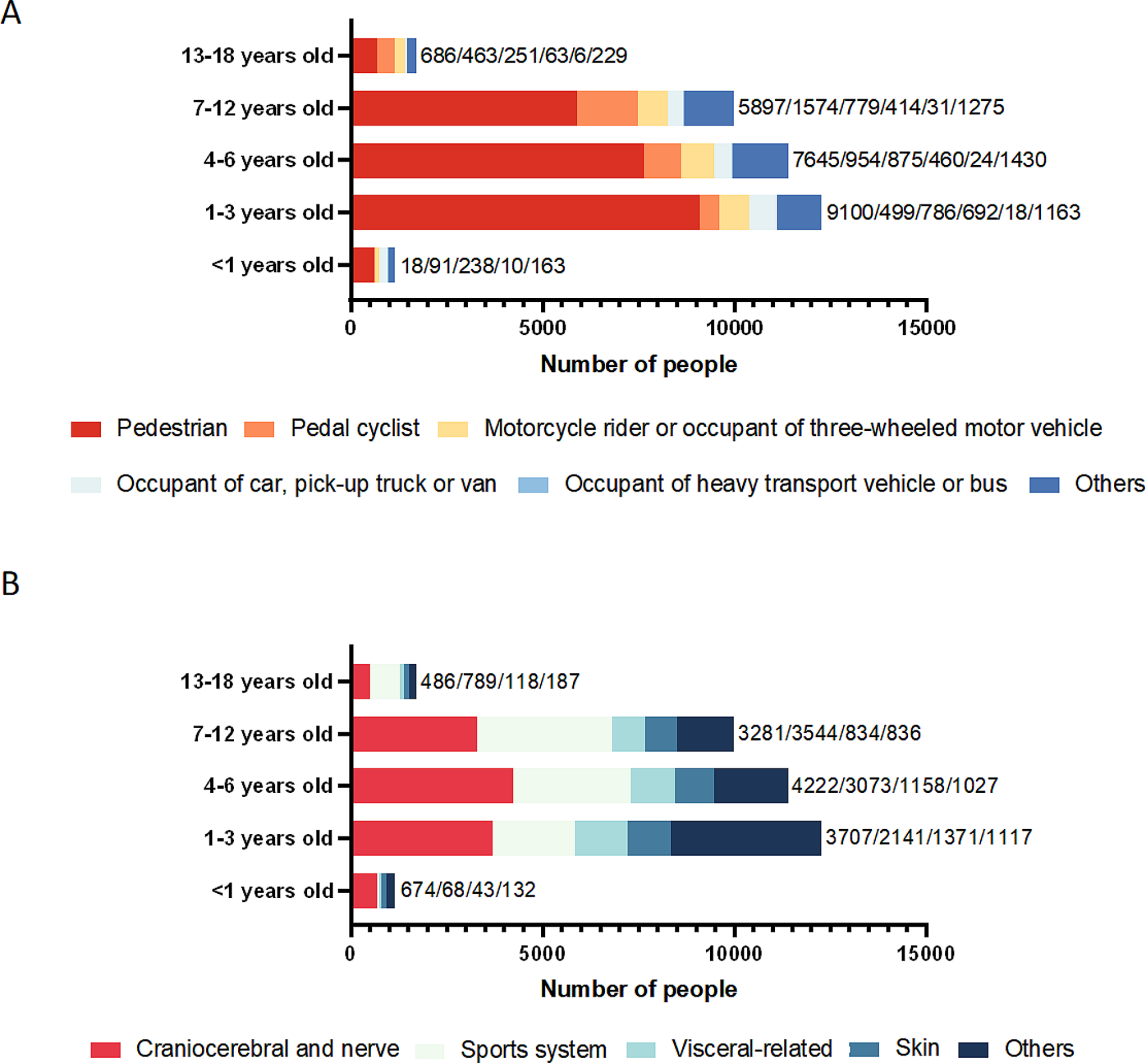
Age distribution of pediatric inpatients in different type (A) and lesion (B) of injured person groups

Our findings confirmed that among 36,455 hospitalization cases, pedestrians were the most common type of injured person (*n* = 23,949, 65.69%, Table 2). Males with transport accidents were markedly more than females in all type of injured person groups (Fig. 3A and Sup Table 3). Except for the types of injured persons < 1 year old were mainly motorcycle rider or occupant of three-wheeled motor vehicle (*n* = 238), the other four age groups were mainly pedestrians (1–3 years old/*n* = 3707, 4–6 years old/*n* = 4222, 7–12 years old/*n* = 3544, 13–18 years old/*n* = 789, Fig. 4A and Sup Table 3). In the type of injured person group, pedal cyclists more susceptible to sport system injury (38.88%). On the other hand, pedestrian (30.21%), motorcycle rider or occupant of three-wheeled motor vehicle (51.26%), occupant of car, pick-up truck or van (50.78%), occupant of heavy transport vehicle or bus (39.33%) were mainly lead to craniocerebral/nerve injury (Table 3). Moreover, the type of injured person were significantly different with the gender (χ^2^ = 88.38, *p* < 0.001), age (χ^2^ = 2677.02, *p* < 0.001), lesion of injured person (χ^2^ = 1702.32, *p* < 0.001) in pediatric inpatients.

**Table 3:** The type of injured person of pediatric inpatients in transport accidents

	Pedestrian	Pedal cyclist	Motorcycle rider or occupant of three-wheeled motor vehicle	Occupant of car, pick-up truck or van	Occupant of heavy transport vehicle or bus	Others	χ2		*P*
**Lesion of person injured**							1702.32		< 0.001
Craniocerebral/nerve	7235 (30.21%)	1119 (31.90%)	1426 (51.26%)	948 (50.78%)	35 (39.33%)	1607 (37.72%)			
Sports system	6092 (25.44%)	1364 (38.88%)	521 (18.73%)	392 (21.00%)	18 (20.22%)	1228 (28.83%)			
Visceral-related	2273 (9.49%)	303 (8.64%)	333 (11.97%)	202 (10.82%)	9 (10.11%)	404 (9.48%)			
Skin	2109 (8.81%)	338 (9.64%)	258 (9.27%)	103 (5.52%)	12 (13.48%)	410 (9.62%)			
Others	6240 (26.06%)	384 (10.95%)	244 (8.77%)	222 (11.89%)	15 (16.85%)	611 (14.34%)			

Of all known lesions, craniocerebral/nerve injury was the more common results in pediatric inpatients in transport accidents (*n* = 12,370, 33.93%, Table 2), and the number of male patients is higher in all kinds of injuries (Fig. 3B and Sup Table 4). Specifically, children younger than 1 year old (*n* = 674), 1–3 years old (*n* = 3707), and 4–6 years old (*n* = 4222) were the most likely to cause craniocerebral/nerve injury, and children 7–12 years old (*n* = 3544) and 13–18 years old (*n* = 789) are the most likely to cause sports injury (Fig. 4B and Sup Table 4). Furthermore, the lesion of injured person was significantly different with the gender (χ^2^ = 26.57, *p* < 0.001) and age (χ^2^ = 2623.64, *p* < 0.001) of patients.

In addition, Table 2 showed the number of children who were discharged in different ways, among which the vast majority of pediatric inpatients were discharged with doctor’s advice (*n* = 32,907, 90.27%). Furthermore, the type of discharge were significantly different with the type of injured person (χ^2^ = 316.27, *p* < 0.001) and lesion of injured person (χ^2^ = 713.24, *p* < 0.001) in pediatric inpatients (Sup Table 5). Specially, occupant of heavy transport vehicle or bus (2.25%) and people with craniocerebral/nerve injury (0.97%) respectively had the highest mortality rate after hospitalization in all type and lesion of injured person groups (Sup Table 5).

### Hospitalization burden of pediatric inpatients in transport accidents

With the benefit of this multi-center database, we were able to better understand the average hospitalization burden of children after traffic accidents. As shown in Table 4, the largest hospitalization burden (included LOS and hospitalization expenses) in the type of injured person was occupant of heavy transport vehicle or bus. Interestingly, among the different lesions of injured person, the hospitalization expense was highest in sports system group, while the LOS was the longest in the visceral-related group. In the end, our results found important differences in the hospitalization burden of patients stratified by the type and lesion of injured person groups.

**Table 4:** The length of hospital stay (LOS) and hospitalization expenses of pediatric inpatients in transport accidents

	Hospitalization expense	χ2	*P*	LOS	χ2	*P*
**Type of injured person**		174.72	< 0.001		378.06	< 0.001
Pedestrian	8650.81 (5059.37, 18782.70)			8 (4, 13)		
Pedal cyclist	10383.38 (5564.59, 17428.19)			6 (4, 10)		
Motorcycle rider or occupant of three-wheeled motor vehicle	10098.17 (5230.58, 21239.64)			9 (5, 14)		
Occupant of car, pick-up truck or van	11351.33 (5500.80, 24873.17)			9 (6, 16)		
Occupant of heavy transport vehicle or bus	11993.41 (4686.71, 23980.36)			9 (5, 16)		
Others	10979.34 (5441.83, 22908.04)			8 (5, 14)		
**Lesion of injured person**		2202.28	< 0.001		2177.64	< 0.001
Craniocerebral/nerve	8745.86 (4789.01, 19110.85)			9 (5, 14)		
Sports system	14334.16 (7689.97, 23918.94)			8 (5, 13)		
Visceral-related	11398.64 (6486.33, 20538.07)			11 (7, 16)		
Skin	7283.65 (4318.66, 11949.20)			6 (4, 10)		
Others	6215.51 (4558.45, 12703.67)			5 (4, 11)		

## Discussion

With the continuous development of the automobile industry and the increasing diversification of transportation modes, traffic accidents have long been a global health challenge. As the leading cause of death for children over the age of five, traffic accidents place a huge physical, psychological and financial burden on children and families [4–6]. In China, the risk of children being injured in traffic accidents is also increasing rapidly, and it has become the second leading cause of death for children aged 1–10 and the first leading cause of death for children aged 15–18 [7]. However, to date, only a few studies have reported on the epidemiological characteristics and hospitalization burden of children suffer from traffic accidents.

Based on a large-scale and multi-center FRCPD database, we reported a national cross-sectional study on the hospitalization information and financial burden of pediatric inpatients in transport accidents, aiming to increase people’s attention to children’s traffic accidents and provide clinical basis for the diagnosis and treatment of children’s traffic accidents. Our results showed that the incidence of road traffic injuries among children in North and East China was higher than in other regions of China. The rate of injury was higher among boys than girls, and among children who had not yet attended kindergarten compared to those in other grades. The skull, brain and nervous system was the most vulnerable body part for younger children. When road traffic injuries occurred, walking was the primary activity for children. Additionally, our results found important differences in the hospitalization burden of patients stratified by the type and lesion of injured person groups.

First, our study also found that boys accounted for the majority of pediatric inpatients in traffic accidents (1.68:1). Similarly, a previous report indicated that boys are more likely to be injured in traffic accidents than girls (1.76: 1) [8]. The possible reason may be that boys are more active, faster, and less caution than girls. Second, given the age of the patients, our results found that children aged 1–3 years were more easily to suffer traffic accidents. Children under 3 years of age do not receive adequate road safety education, due to they are not enrolled in the kindergarten. Therefore, the incidence of traffic accidents will increase when children with poor mobility and judgment ability are walking, playing, and crossing the road [9]. In addition, the number of children aged < 1 or 13–18 in traffic accidents obviously decreased compared to other age groups in our study. Children < 1 year old can’t walk very well, and are generally looked by caregivers for a long time. Children aged 13–18 have better safety awareness, and children > 14 years old are often admitted to adult hospitals in China [8, 10]. In addition, the number of children hospitalized due to traffic accidents peaked in July. Similarly, a study reported that the hospitalization peak of pediatric traumatic brain injury was the third quarter of the year, which may be related to children’ easier outdoor activities during the summer vacation [15].

Then, the number of hospitalization children in traffic accidents were significantly higher in North and East China than in other regions in the current study. On the one hand, many scholars believe that the high degree of urbanization, large population density and large vehicle flow are the risk factors that lead to frequent traffic accidents [11]. Due to the economic conditions, terrain characteristics and other factors in different regions of a country, the incidence and death rate of traffic accidents are also different [2]. Thus, as the economically developed regions, North and East China have higher incident of traffic accidents [11, 13, 14]. On the other hand, a previous study found that as the number of health facilities increased, the number of deaths from traffic accidents decreased, so the investment of health facilities located in the area needed to be improved [12]. Thus, a large number of patients will also be treated and transferred to North and East China, due to the backward medical resources in other regions. Unfortunately, this FRCPD database does not cover and extract the hospitalization information of children’s hospitals such as Guangzhou and Shanghai, so the regions analysis remain limitations.

Next, our study found that pedestrian was the most common type of injured person in the FRCPD database. Possible explanations for this results were that active children are unable to judge the distance and speed of cars approaching them in a timely and accurate manner, and the parents often ignore the dangers of their children as pedestrians [11]. Furthermore, the patients with craniocerebral/nerve injury were mainly 4–6 years old, the patients with sport injury were 7–12 years old and 13–18 years old, and the patients with visceral-related or skin injury were 1–3 years old. In addition to pedal cyclists more susceptible to sport system injury, other types of injured person with transport accidents were mainly craniocerebral/nerve injury in our results. Past studies have reported that children on the road are usually impacted by the front end of the vehicle, and the head and lower extremity are the most vulnerable areas to suffer moderate and severe injuries [13, 14]. In motor vehicle collisions, head injuries are most common in children, which is independent of the age of the child, whether the child was restrained by a seat belt, or crash direction [15–17]. Body proportions of children change dramatically throughout the growing period. The head accounts for about a quarter of the body length at birth, and only about 1/7 of the body length in adulthood [18]. A study has found that children under the age of 6 are more likely to suffer craniocerebral/nerve injury [17]. This may be related to the higher head weight of children in this age group, and the head is more likely to hit the ground first in traffic accidents, resulting in craniocerebral/nerve injury. With the increase of children’s age, the proportion of craniocerebral/nerve injury gradually decreased, but sport system injuries increased correspondingly. Adolescents have an increased subconscious self-protection reflex in dangerous, and they often use their limbs for emergency protection, which leads to an increased possibility of damage to the sport system. A cohort study has confirmed that the extremities (47.7%) are the most likely to be injured in bicycle-related trauma [19].

The hospitalization burden of traffic accidents (including LOS and hospitalization expenses) will have a huge impact on the families and society. Therefore, this paper analyzed the factors affecting the hospitalization burden of children of traffic accidents. Among the different lesions of injured person in our study, the hospitalization expense was highest in sports system group, while the LOS was the longest in the visceral-related group. In traffic accidents, children with visceral-related injury often have visceral rupture, so clinical treatment such as indwelling drainage tube is needed. Patients with these injuries generally need to stop eating and drinking for a long time, and the recovery process may be prolonged, ultimately resulting in a longer LOS. Additionally, hospitalization costs include treatment, drugs, materials, tests, care and other costs. For those with sports injuries, surgery often involves the use of expensive medical materials, such as fixation frames and Kirschner wires, which can significantly increase the expenses of hospitalization.

## Conclusion

This study used a relatively large-scale, multi-center representative sample from China in recent years, presenting evidence-based epidemiological characteristics and hospitalization burden for pediatric inpatients in transport accidents. Therefore, the prevention of road traffic injuries among children in China remains urgent and requires a multi-pronged approach. Publicity and enforcement of traffic laws should be strengthened, especially in the eastern and northern regions. It is proposed to add helmets for cyclists to traffic regulations, strengthen traffic safety education starting from kindergartens, and strengthen penalties for pedestrians violating traffic safety laws.

### Electronic supplementary material

Below is the link to the electronic supplementary material.

Supplementary Material 1

## Data Availability

The original data presented in the study are included in the article material, further inquiries can be directed to the corresponding authors.

## Abbreviations

FRCPD: The Futang Research Center of Pediatric Development
WHO: World Health Organization
HQMS: Hospital Quality Monitoring System
LOS: Length of hospital stay
