# Supplementary material for: Hospitalization information and burden of pediatric inpatients in transport accidents
Source: BMC Public Health. 2024 May 30;24:1446. doi: 10.1186/s12889-024-18891-2 (PMC11137972; doi:10.1186/s12889-024-18891-2)
Supplement: Supplementary file 1 — Supplementary Material 1 [file 12889_2024_18891_MOESM1_ESM.doc]

**Hospitalization information and burden of** **pediatric inpatients in transport accidents**

**Sup Table 1 The detailed number of people included in each children’s hospitals in our study.**

| **Region** | **Children’s hospitals** | **Number** |
| --- | --- | --- |
| Northeast China | Harbin Children's Hospital | 468 |
|  | Changchun Children’s Hospital | 74 |
|  | Dalian Children's Hospital | 933 |
| North China | Beijing Children's Hospital | 1076 |
|  | Hebei Children's Hospital | 7281 |
|  | Baoding Children’s Hospital | 677 |
|  | Shanxi Children's Hospital | 1217 |
|  | Inner Mongolia Maternity and Child Health Hospital | 7 |
| East China | Jinan Children's Hospital | 637 |
|  | Liaocheng Children's Hospital | 1595 |
|  | Nanjing Children's Hospital | 4077 |
|  | Suzhou University Affiliated Children’s Hospital | 2836 |
|  | Xuzhou Children’s Hospital | 913 |
|  | Anhui Children's Hospital | 2619 |
|  | Hangzhou Children's Hospital | 60 |
|  | Jiangxi Children's Hospital | 2028 |
|  | Fuzhou Children's Hospital | 315 |
| South China | Liuzhou Maternity and Child Health Hospital | 51 |
|  | Shenzhen Children's Hospital | 1474 |
| Central China | Zhengzhou Children's Hospital | 714 |
|  | Wuhan Children's Hospital | 1575 |
|  | Hunan Children's Hospital | 631 |
| Northwest China | Urumqi Children's Hospital | 253 |
|  | Gansu Maternity and Child Health Hospital | 32 |
|  | Qinghai Maternity and Child Health Hospital | 1042 |
|  | Xi’an Children's Hospital | 2177 |
| Southwest China | Guiyang Maternity and Child Health Hospital | 140 |
|  | Kunming Children's Hospital | 1553 |

**Sup Table 2 Specific number of pediatric inpatients in traffic accidents per month, 2016 to 2021.**

|  | **2016** | **2017** | **2018** | **2019** | **2020** | **2021** |
| --- | --- | --- | --- | --- | --- | --- |
| **January** | 375 | 435 | 335 | 419 | 296 | 253 |
| **February** | 452 | 470 | 465 | 425 | 110 | 335 |
| **March** | 511 | 490 | 522 | 465 | 250 | 389 |
| **April** | 545 | 559 | 617 | 394 | 441 | 496 |
| **May** | 556 | 673 | 678 | 436 | 634 | 573 |
| **June** | 571 | 655 | 635 | 506 | 615 | 577 |
| **July** | 619 | 630 | 646 | 571 | 624 | 637 |
| **August** | 583 | 567 | 617 | 488 | 603 | 610 |
| **September** | 621 | 611 | 673 | 481 | 593 | 635 |
| **October** | 550 | 573 | 661 | 449 | 573 | 537 |
| **November** | 477 | 570 | 491 | 396 | 431 | 414 |
| **December** | 417 | 469 | 406 | 323 | 333 | 418 |

**Sup Table 3 The type of injured person of pediatric inpatients in transport accidents.**

|  | **Pedestrian** | **Pedal cyclist** | **Motorcycle rider or occupant of three-wheeled motor vehicle** | **Occupant of car,** **pick-up truck or van** | **Occupant of heavy transport vehicle or bus** | **Others** | **χ2** | **P** |
| --- | --- | --- | --- | --- | --- | --- | --- | --- |
| **Gender** |  |  |  |  |  |  | 88.38 | <0.001 |
| Male | 15234 | 2338 | 1632 | 1066 | 53 | 2563 |  |  |
| Female | 8715 | 1170 | 1150 | 801 | 36 | 1697 |  |  |
| **Age** |  |  |  |  |  |  | 2677.02 | <0.001 |
| <1 years old | 621 | 18 | 91 | 238 | 10 | 163 |  |  |
| 1-3 years old | 9100 | 499 | 786 | 692 | 18 | 1163 |  |  |
| 4-6 years old | 7645 | 954 | 875 | 460 | 24 | 1430 |  |  |
| 7-12 years old | 5897 | 1574 | 779 | 414 | 31 | 1275 |  |  |
| 13-18 years old | 686 | 463 | 251 | 63 | 6 | 229 |  |  |

**Sup Table 4 The lesion of injured person of pediatric inpatients in transport accidents.**

|  | **Craniocerebral and nerve** | **Sports system** | **Visceral-related** | **Skin** | **Others** | **χ2** | **P** |
| --- | --- | --- | --- | --- | --- | --- | --- |
| **Gender** |  |  |  |  |  | 26.57 | <0.001 |
| Male | 7606 | 6232 | 2205 | 2007 | 4836 |  |  |
| Female | 4764 | 3383 | 1319 | 1223 | 2880 |  |  |
| **Age** |  |  |  |  |  | 2623.64 | <0.001 |
| <1 years old | 674 | 68 | 43 | 132 | 224 |  |  |
| 1-3 years old | 3707 | 2141 | 1371 | 1117 | 3922 |  |  |
| 4-6 years old | 4222 | 3073 | 1158 | 1027 | 1908 |  |  |
| 7-12 years old | 3281 | 3544 | 834 | 836 | 1475 |  |  |
| 13-18 years old | 486 | 789 | 118 | 118 | 187 |  |  |

**Sup Table 5 The type of discharge of pediatric inpatients** **in transport accidents.**

|  | **Discharge with doctor’s advice** | **Discharge without doctor’s advice** | **Death** | **Transfer with doctor’s advice** | **Transfer to community health service agencies/township health center with doctor’s advice** | **Others** | **χ2** | **P** |
| --- | --- | --- | --- | --- | --- | --- | --- | --- |
| **Type of injured person** |  |  |  |  |  |  | 316.27 | <0.001 |
| Pedestrian | 21312 | 2299 | 109 | 36 | 22 | 171 |  |  |
| Pedal cyclist | 3330 | 149 | 4 | 8 | 2 | 15 |  |  |
| Motorcycle rider or occupant of three-wheeled motor vehicle | 2497 | 210 | 14 | 10 | 3 | 48 |  |  |
| Occupant of car, pick-up truck or van | 1714 | 110 | 16 | 8 | 1 | 18 |  |  |
| Occupant of heavy transport vehicle or bus | 83 | 2 | 2 | 0 | 0 | 2 |  |  |
| Others | 3971 | 196 | 26 | 11 | 1 | 55 |  |  |
| **Lesion of injured person** |  |  |  |  |  |  | 713.24 | <0.001 |
| Craniocerebral/nerve | 11088 | 946 | 120 | 39 | 5 | 172 |  |  |
| Sports system | 9081 | 467 | 5 | 9 | 18 | 35 |  |  |
| Visceral-related | 3288 | 181 | 14 | 10 | 0 | 31 |  |  |
| Skin | 2849 | 357 | 2 | 4 | 1 | 17 |  |  |
| Others | 6601 | 1015 | 30 | 11 | 5 | 54 |  |  |
